# Spatial Scene Memories Are Biased Towards a Fixed Amount of Semantic Information

**DOI:** 10.1162/opmi_a_00088

**Published:** 2023-07-21

**Authors:** Michelle R. Greene, Devanshi Trivedi

**Affiliations:** Bates College, Program in Neuroscience; Barnard College, Columbia University; Oxford University

**Keywords:** boundary extension, scene perception

## Abstract

Scene memory has known spatial biases. Boundary extension is a well-known bias whereby observers remember visual information beyond an image’s boundaries. While recent studies demonstrate that boundary contraction also reliably occurs based on intrinsic image properties, the specific properties that drive the effect are unknown. This study assesses the extent to which scene memory might have a fixed capacity for information. We assessed both visual and semantic information in a scene database using techniques from image processing and natural language processing, respectively. We then assessed how both types of information predicted memory errors for scene boundaries using a standard rapid serial visual presentation (RSVP) forced error paradigm. A linear regression model indicated that memories for scene boundaries were significantly predicted by semantic, but not visual, information and that this effect persisted when scene depth was considered. Boundary extension was observed for images with low semantic information, and contraction was observed for images with high semantic information. This suggests a cognitive process that normalizes the amount of semantic information held in memory.

## INTRODUCTION

It is said that pictures are worth one thousand words. If so, how many of these words can we retain in memory after viewing a scene? Although scene memory is excellent (Brady et al., 2008), these memories come with specific biases. One’s scene memories can contain more space than the picture depicted (boundary extension; Intraub & Richardson, 1989), or they may be smaller (boundary contraction; Bainbridge & Baker, 2020a). A given scene will consistently expand or contract in observers’ memories, yet the scene properties that lead to one type of scene transformation have not yet been fully characterized. This work approaches the question from the perspective of information capacity limitations. Here, we ask whether there is a bias towards storing constant amount of scene *information* in visual memory. If this is the case, scenes that exceed the information capacity limit will contract, while scenes under the information capacity limit will expand. We measured the relative amounts of visual and semantic information from scenes in a large scene database and assessed the boundary transformations for each scene. We found that a scene’s semantic information predicted boundary transformation scores in the manner predicted by the fixed information hypothesis.

Initial studies of spatial biases in scene memory reported only boundary extension (Intraub, 2012; Intraub & Richardson, 1989). However, it was a remarkably consistent effect—it was observed across retention intervals ranging from milliseconds to days, across different testing modalities, and across observers ranging in age from childhood to late adulthood (for a review, see Hubbard et al., 2010). More recent work has demonstrated that in a larger and more diverse scene database, boundary extension and contraction are equally likely to occur (Bainbridge & Baker, 2020a). Although ongoing discussion is still establishing why the older image sets only produced boundary extension (Bainbridge & Baker, 2020b; Intraub, 2020), several recent studies have replicated and extended the basic finding of bidirectional boundary transformations (Hafri et al., 2022; Lin et al., 2022; Park et al., 2021).

In these studies, there is general agreement that scene depth plays a pivotal role. Bainbridge and Baker (2020a) noted that scene composition was a significant predictor of the effect: images composed of a central object in a relatively homogeneous background consistently led to boundary extension, while more expansive scenes led to boundary contraction. After creating a set of 3D rendered environments that spanned the object-to-scene continuum, Park et al. (2021) found that larger depth was associated with more boundary contraction but that no absolute viewing distance was associated with the change between boundary extension and contraction. Rather, the transition point was scene-specific and biased towards common, canonical views of the given environment. Congruently, Lin et al. (2022) found that spatial scene memories were biased towards the modal depth for a given scene category, using images from a large scene database with measured depth values.

A second emerging hypothesis is that image properties that provide depth cues may be causally driving the effect. Camera aperture settings that are correlated with scene scale are highly predictive of boundary transformations (Gandolfo et al., 2022). In a similar vein, Hafri et al. (2022) used image processing techniques to manipulate the perceived depth of images. They found that expansive images that were made to appear small led to less boundary contraction, consistent with the idea that image features that give rise to spatial scale drive boundary transformations rather than knowledge of scene categories and their expected mean depth values.

There are also reasons to believe that scene depth may not be the sole feature responsible for boundary transformations. Scenes that produce the most boundary extension are relatively sparse, such as a central object on a simple background (Bainbridge & Baker, 2020a). This suggests that scenes with similar depth may produce different boundary transformations depending on the richness of their content. Images with highly emotional content can produce boundary contraction (Takarangi et al., 2016), suggesting that specific image content can drive boundary transformations. The goal of this study is to assess whether increases in either semantic or visual information play a role in scene boundary transformations.

The present research measures scene information and tests the extent to which information predicts scene transformation scores. As scenes contain both visual and semantic information, we used image processing techniques to measure visual information and natural language processing to measure semantic information. Using a standard RSVP boundary extension paradigm, we show that a scene’s semantic information significantly predicts scene boundary transformations over and above the effect of scene depth. Specifically, images with low semantic information tend to expand their boundaries, while images with high semantic information contract. Altogether, this process suggests a scene memory representation that is normalized towards a fixed amount of semantic information.

## MATERIALS AND METHODS

### Stimuli

We used 1000 images from recent work on boundary extension (Bainbridge & Baker, 2020a). This dataset includes 500 scene-centric images from the SUN database (Xiao et al., 2014) and 500 object-centered images from the Google Open Images Dataset (Kuznetsova et al., 2020). Each image was center cropped and resized to 350x350 pixels. We computed information measures on visual and semantic features (see below) on all 1000 images and then selected 120 images that spanned the range of both information metrics. Of these, 68 were from the Google Open Images Dataset, and 52 were from the SUN database.

### Measuring Visual Information

Our goal was to compare the amount of visual information across scene images. Although there have been efforts to estimate the entropy of natural scenes based on patches of natural images (Chandler & Field, 2007; Hosseini et al., 2010; Petrov & Zhaoping, 2003), the number of images and computational resources needed to estimate the entropy of natural scenes has remained intractable fully. Therefore, we aim to estimate the *relative* amount of visual information within this dataset rather than computing a specific number of bits for each image.

**Figure 1.**
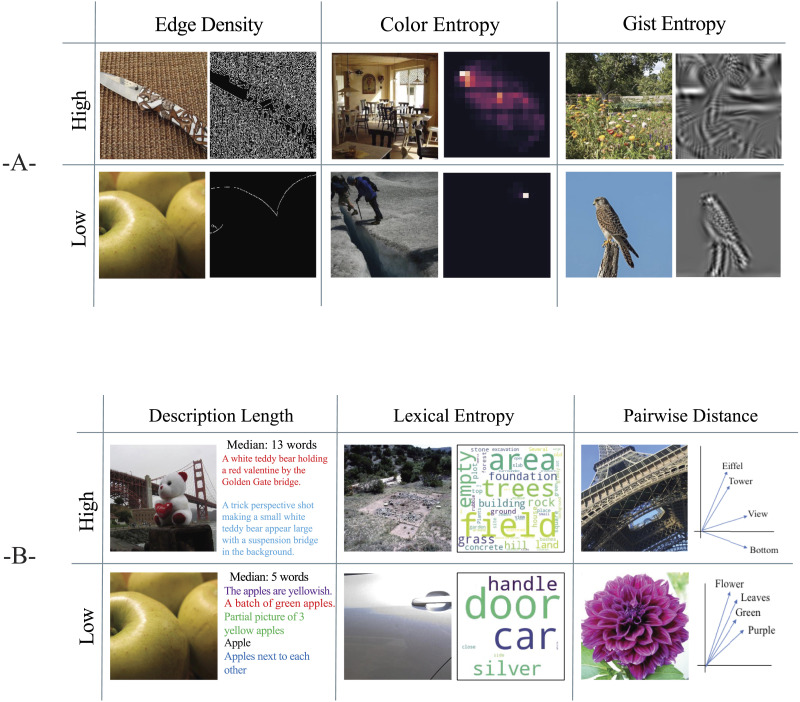
**(A) Examples of the three visual information features.** For edge density, we summed the number of pixels containing edges. Color variability was assessed via the entropy of A*-B* histograms. The entropy of the Gist description provided a metric of the variability of oriented edge content at various spatial scales and image locations. For each, the lowest and highest values are shown, along with a visualization of the feature. (B) Examples of the three semantic information features. To illustrate description length, we provide several example descriptions for the longest and shortest descriptions. Lexical entropy is visualized by word clouds. Lexical entropy increases when the number of unique words increases. The pairwise similarity is visualized using a simplified 2D graph as a stand-in for the high-dimensional vector space representing each word. A small average cosine distance was obtained for related concepts. For each, the lowest and highest values are shown, along with a visualization of the feature.

Because there is no single way to compute visual information, our approach was to compute three measures that describe different aspects of scene information and complexity and then employ dimensionality reduction (principal components analysis (PCA)) to unify what is common in these metrics into one measure of visual information. Although some recent work has employed similar features and a whitening transformation to reduce multicollinearity (Greene & Hansen, 2020), we decided to use PCA to unify the metrics because we had no specific hypotheses about the individual metrics and instead wanted an aggregate measure of visual information. Each of the three features is shown visually in Figure 1A.

The first measure was edge density from a Canny edge detector. Intuitively, an image with more contours will have more complexity and, therefore, more information. Edge density has been shown to correlate with observers’ subjective assessments of image complexity (Ciocca, Corchs, & Gasparini, 2015a; Corchs et al., 2016; Nagle & Lavie, 2020), and eye fixations tend to land on areas of higher edge density (Parkhurst & Niebur, 2003) suggesting that observers need to foveate these regions to disambiguate their higher information content. Each image was converted to grayscale. A 5-pixel by 5-pixel Gaussian blur was applied to the image to reduce pixel noise. The lower and upper bounds for hysteresis thresholding were 30 and 150 pixels, respectively. The number of pixels that corresponded to edges was stored for each image.

The second metric described how colorful a scene was by computing the entropy of the image’s color histogram. A similar metric correlates with subjective ratings of colorfulness (Hasler & Suesstrunk, 2003). The role of colorfulness in image complexity and information has had mixed results. In a study of abstract textures, images with a greater variety of colors were rated as more complex (Kocaoğlu & Olguntürk, 2019). However, images of real-world scenes received similar complexity ratings in both color and grayscale versions (Ciocca, Corchs, Gasparini, et al., 2015b), suggesting that color plays little role in the assessment of subjective visual complexity. Each image was converted from RGB to CIELAB color. Using the A* and B* color channels, we created a two-dimensional histogram with 20 bins each. The value stored in each histogram cell reflected the number of pixels in the given A*-B* color range. We converted this histogram to a probability distribution and computed and stored its entropy. Entropy is a measurement of uncertainty (Shannon, 1948) and is computed with the following formula:

$$H=-1\;\sum_{i=1}^{n}p_{i}\log_{2}p_{i}$$

where *n* is the number of bins in the discrete probability distribution, and *p*_*i*_ is the probability associated with the ith bin. Entropy is maximized for uniform distributions and approaches 0 as any *p*_*i*_ approaches 1.

To choose the bin size, we selected a separate set of six images containing three images with subjectively low color variability and three images with subjectively high color variability. We computed color histograms with bin sizes between 2 and 200 and examined the entropy differences between the two image groups. We found that a bin size of 20 provided the largest and most reliable differences between the two groups.

The third metric was entropy in the Gist descriptor of Oliva and Torralba (2001). This metric describes the dominant orientations and spatial frequencies at different image locations. This descriptor has been shown to be helpful in scene classification (Oliva & Torralba, 2001) and correlates with neural responses to scenes (Watson et al., 2017). Here, we used three spatial scales with 8, 6, and 4 orientations per scale at each of the 64 image locations for a total of 1152 features. We normalized the response vector to sum to 1 and computed entropy over this representation.

Thus, each image’s visual information was represented by three numbers: edge density, color entropy, and gist entropy. We computed principal components analysis (PCA) on this matrix and projected all images onto the first principal component to create a unified measure of visual information. This component accounted for 97% of the variance in the original data.

### Measuring Semantic Information

While the visual information metrics describe the quantity and variety of physical image features, we would also like to describe the amount of meaning in an image as well. These cannot be computed directly from the image, so we conducted a preliminary study to obtain observers’ descriptions of images. From these, we used natural language processing to compute several metrics of the complexity of these descriptions. We will detail each of these below; see Figure 1B for visual descriptions.

For the description experiment, 1112 participants on Amazon’s Mechanical Turk (mTurk) platform viewed trials in which they were shown one of the 1000 photographs and asked to describe the image such that another person could pick it out of a lineup of similar images. We restricted the experiment to US-based persons who had previously completed at least 1000 previous hits with at least 95% accuracy. Each individual could complete as many of the 1000 trials as desired. The median number of trials per participant was 10 (range: 1 to 1000).

As with visual information, we extracted three features that reflected semantic information in these descriptions and used PCA to combine them into one unified measurement. The first metric was each image’s median description length (in words). We reasoned that participants’ image descriptions followed the Gricean maxim of informativeness—that is, they only contained content that would not be taken for granted by a reader (Grice, 1991). Thus, all things being equal, images that consistently elicited longer descriptions contained more semantic information than those with shorter descriptions. High-frequency words (Piantadosi et al., 2011) and words with highly predictive contexts (Mahowald et al., 2013) tend to be shorter, reflecting communication strategies that maximize information transmission.

The second metric was the entropy of a bag-of-words description (Wang et al., 2020). We used the NLTK python library (Bird et al., 2009) to convert each description to lowercase and to remove punctuation and the default stopwords from the nltk library (common non-content words such as ‘the’, ‘and’, ‘I’). From these, we created a dictionary of all unique word tokens (a total of 19,556 words across the 100,000 unique descriptions). For each image, we created a histogram of word token use across the 100 descriptions and computed entropy over this histogram. Under this metric, an image with more varied descriptions across observers or more unique work tokens will receive a higher entropy measurement. This metric has been shown to correlate with subjective complexity ratings of abstract art (Wang et al., 2020).

The last metric we computed was the mean semantic distance between pairs of words as evaluated by a Word2vec, a word embedding model (Mikolov et al., 2013) trained on the Google News dataset. The logic behind this metric is that larger distances between words will indicate less standard context and, thus, more semantic information. Encoding models based on word2vec have shown to be helpful in predicting neural responses in object-selective cortex (Bonner & Epstein, 2021). For each description, we removed stopwords using NLTK (Bird et al., 2009). With the remaining words, we computed the cosine distance between each pair of remaining word tokens. For example, for the description “The quick brown fox jumps over the lazy dog”, we would have the following tokens after omitting stopwords: “Quick brown fox jumps over lazy dog”. These seven words contain 21 unique word pairs, and cosine distance was computed between each pair (for example, quick-brown, quick-fox, quick-jumps, etc.). We computed the mean similarity across all distance pairs and across the 100 descriptions for each image. High similarity scores are obtained when two words appear close together in language contexts.

Thus, each image’s semantic information was represented by three metrics: median description length, average lexical entropy, and average pairwise similarity. We performed PCA on this matrix and projected each image onto the first principal component for a single metric of semantic information. This first component explained about 86% of the original variability across images.

### Comparing Image Metrics

**Figure 2.**
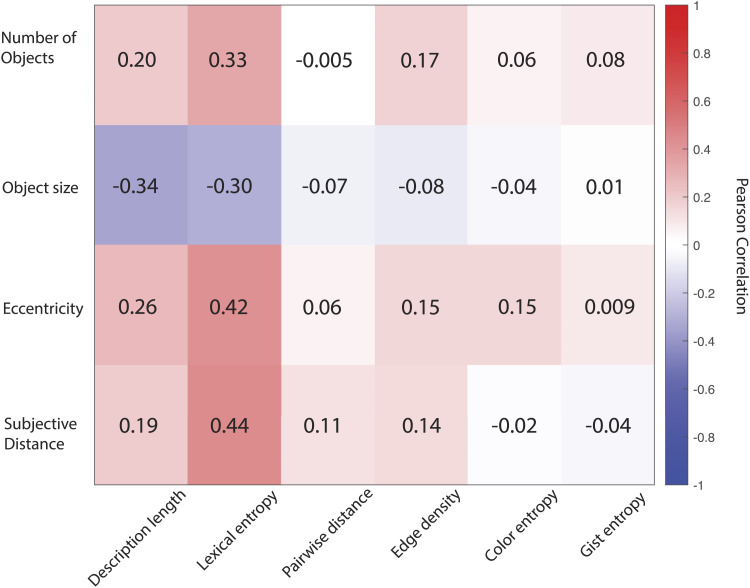
Pearson correlation coefficients between the image metrics from Bainbridge and Baker (2020a), shown in rows, and our semantic and visual information metrics shown in columns.

In order to compare our semantic and visual information metrics to existing metrics on the same images, we computed correlation coefficients between each of the four metrics measured by Bainbridge and Baker (2020a) and our metrics. As shown in Figure 2, Pearson correlation coefficients were low to moderate across the board. We observed higher absolute correlations between Bainbridge and Baker’s metrics and our semantic information metrics, compared to our visual information metrics. Further, the highest absolute correlations with the Bainbridge and Baker metrics were found between lexical entropy, which was positively correlated with the number of objects, object eccentricity, and subjective scene distance, and negatively correlated with object size.

### Experimental Procedure

We assessed boundary transformations using a standard rapid serial visual presentation (RSVP) paradigm (Bainbridge & Baker, 2020a; Hafri et al., 2022; Lin et al., 2022; Park et al., 2021). We recruited 360 US-based participants on the online experiment platform Prolific (mean age = 38 years, 288 female, 61 male, 11 nonbinary). The experiment was approved by the Bates College IRB, and volunteers provided written informed consent before participating. Our sample size was determined via a power analysis (conducted in the pwr library of R) using the effect size reported in (Bainbridge & Baker, 2020a). Our sample size is in line with Hafri et al. (2022).

**Figure 3.**
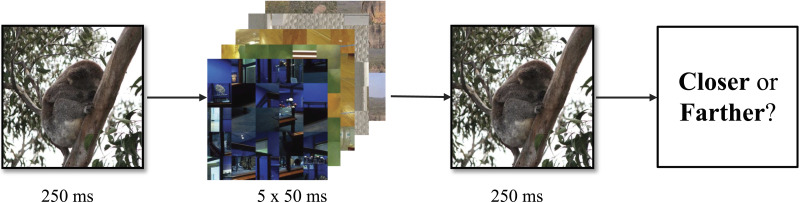
**Trial structure for the experiment.** We employed a forced error paradigm in which observers were asked to indicate whether the second presentation of an image was closer or farther than the originally-presented image after a dynamic mask.

Each participant viewed each of the 120 images in random order. The trial structure for this experiment is shown in Figure 3. Each of the 120 trials consisted of the target image shown for 250 ms, followed by a rapid serial visual presentation (RSVP) stream of five block-scrambled masks shown for 50 ms each. None of the mask images were from the experimental image set. Following the mask series, participants were presented with the same target image for 250 ms and asked to assess whether the second version was more contracted (zoomed in) or expanded (zoomed out) compared to the previous image. As the two images were identical, participants were making a forced error. For this reason, no performance feedback was provided.

If there were no systematic memory biases for scene boundaries, we would expect a roughly equal number of people to make each error type. If boundary extension is the primary mode of boundary transformation, we would expect participants to indicate that the second presentation of each image was more contracted (zoomed in). However, if some images are prone to boundary extension and others to boundary contraction, we should see systematic biases in both directions but differing by image, as found by Bainbridge and Baker (2020a). This experiment tests the hypothesis that memory for scene boundaries might have a fixed information capacity. Thus, we predicted that images with more information would lead to contraction, while images with less information would lead to extension.

## RESULTS

We correlated the visual and semantic information metrics to assess their relative independence. Across the 120 images in this experiment, we found no significant correlation between them (*r* = 0.03, *t*(118) = 0.31, *p* = 0.76). Therefore, these two metrics tap into relatively independent sources of image information.

For each of the 120 images, we computed a boundary transformation score across the 360 observers. This score was the average of the number of observers who made an extension error (coded as +1) to the number of observers who made a contraction error (coded as −1). Thus, scores larger than 0 indicate boundary extension, while negative scores indicate boundary contraction. If no systematic tendency is observed, this score would be about 0.

Boundary transformation scores ranged from −0.22 to 0.55. 97 images (81%) showed boundary extension, while 22 images showed boundary contraction, and one image had a transformation ratio of 0.0. Our results were highly similar to the results obtained by Bainbridge and Baker (2020a): our scores were highly correlated with their boundary transformation scores (*r* = 0.86, *t*(118) = 17.93, *p* < 2.2e−16). Thus, this work also provides an independent replication of their work.

To compute the noise ceiling of the data, we estimated how well half of the participants’ boundary transformation scores predicted the scores of the other participants. In 10,000 iterations, we randomly split participants into two groups and computed the boundary transformation scores for each group, and then predicted the first scores with the second via linear regression. We found that 95% of the splits had *R*^2^ values between 0.69 and 0.79. This sets an upper bound to how well any model can predict boundary transformation scores.

Finally, we computed a baseline model with only depth (rankings provided by Bainbridge and Baker (2020a) predicting boundary transformation scores. A significant regression equation was found (*F*(1, 116) = 210.3, *p* < 2.2e−16) with an *R*^2^ of 0.64. In other words, increasing the mean depth of an environment decreases the probability that the scene’s boundaries will expand in memory, as has been found in other recent work (Bainbridge & Baker, 2020a; Hafri et al., 2022; Park et al., 2021).

Our main hypothesis is that the information contained in visual memory is relatively constant. This would predict that images with too much information will contract in memory while images with too little information will expand. We tested this through multiple linear regression analysis, including the visual or semantic information scores as additional predictors for the boundary transformation score of each image.

When semantic information was added to the model, a significant regression equation was found (*F*(2, 115) = 123.4, *p* < 2.2e−16) with an adjusted *R*^2^ of 0.68 (99% of the lower bound of the noise ceiling). However, when visual information was added to the depth model, we did not observe an increase in *R*^2^ over the baseline depth model (*F*(2, 115) = 105, *p* < 2.2e−16, adjusted *R*^2^ = 0.64). Similarly, a model with all three predictors had a sightly lower adjusted *R*^2^ than the model containing only semantic information and depth (*F*(3, 114) = 82, *p* < 2.2e−16, adjusted *R*^2^ = 0.68, see Table 1). Therefore, visual information does not contribute significantly to memory distortions of scene boundaries.

**Table 1.** Regression coefficients and statistics.

	**Coefficient**	***t*-value**	***p*-value**
Intercept	0.52	20.6	2e−16
Depth	−0.13	−14.8	2e−16
Semantic Information	−0.03	−3.70	0.0003
Visual Information	−0.001	0.732	0.47

In order to understand which semantic features drove this result, we ran separate regression analyses for each of the three semantic features. We found that median description length was a significant predictor of boundary transformation scores (*β* = −0.06, *F*(1, 118) = 18.99, *p* = 2.83e−05, *R*^2^ = 0.13, as was the lexical entropy measurement (*β* = −0.23, *F*(1, 118) = 30.6, *p* = 1.9e−7, *R*^2^ = 0.20). However, we did not find mean pairwise semantic similarity to be a significant predictor of boundary transformation scores (*F*(1, 118) < 1). Therefore, images that elicited longer descriptions and also descriptions that were more variable across observers were associated with boundary contractions, while images that elicited shorter and more homogeneous descriptions were associated with boundary extension.

**Figure 4.**
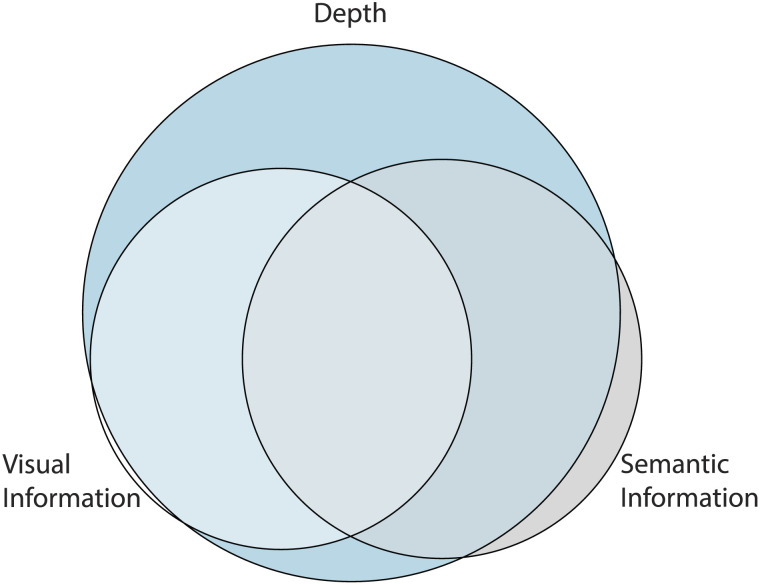
**Euler diagram of variance partition analysis.** The size of the ellipses are proportional to the explained variance of each model, and the degree of shared variance between model is illustrated with the overlap of ellipses.

The previous results indicated that semantic information provides additional predictive power over and above that provided by a scene’s depth. However, we do not net know if the variability accounted for with semantic information is independent of that accounted for by depth. To address this question, we used depth, semantic, and visual information as predictors to perform a variance partitioning analysis (Greene et al., 2016; Groen et al., 2018; Lescroart et al., 2015). In total, seven regression models were constructed: (1) the three features together; (2–4) each pair of features; (5–7) each individual feature. From these, simple arithmetic calculations on the *R*^2^ values between the models can allow us to infer each model’s shared and independently explained variance. We visualized the result in an Euler diagram (eulerr package, R) in Figure 4. Congruent with our previous analyses, we can see that semantic (but not visual) information provides some additional explained variance for boundary transformation scores.

Therefore, in addition to confirming previous findings that larger depth scenes are associated with boundary contraction instead of extension, our results show that higher semantic content is associated with boundary contraction, even when image depth is held constant.

## DISCUSSION

It is said that pictures are worth one thousand words, but not all pictures evoke similarly rich descriptions. In other words, scenes differ in their semantic visual information. Inspired by recent studies showing that observers’ memories of the spatial boundaries of real-world scenes can either expand or contract (Bainbridge & Baker, 2020a; Hafri et al., 2022; Lin et al., 2022; Park et al., 2021), this paper tested the hypothesis that scene memories are biased towards a fixed amount of information. Using image processing and natural language processing techniques to create proxies for visual and semantic information, we found that semantic (but not visual) information predicted whether a scene’s boundaries would contract or expand in memory and that this relationship held even when scene depth was held constant. Specifically, scenes with less semantic information tended to expand. In contrast, scenes with more semantic information tended to contract, consistent with the hypothesis that the amount of semantic information in visual memory is fixed.

Many recent studies have pointed to the role of scene depth in boundary extension (Bainbridge & Baker, 2020a; Bertamini et al., 2005; Gandolfo et al., 2022; Hafri et al., 2022; Lin et al., 2022; Park et al., 2021). Specifically, scenes with small fields of view tend to produce boundary extension, while panoramic spaces can produce boundary contraction. Using the scene depth ratings provided by Bainbridge and Baker (2020a), we have both replicated and extended their result. We have found that semantic information, in addition to scene depth, predicts the amount of remembered visual space. Scenes with small mean depth also likely limit semantic information, as they tend to depict a single object against a background. However, a scene with a large depth of field does not necessarily have a large amount of semantic information. For example, an empty warehouse may have very little semantic information. Thus, scene depth may be necessary, but not sufficient for boundary contraction, and semantic information may explain why not all large-scale scenes produce boundary contraction or why only boundary extension was previously reported in the literature (Intraub & Richardson, 1989).

There are several theoretical accounts of the boundary extension phenomenon. Early theories linked boundary extension to predictive cognition and mental simulation. According to this account, representing space beyond the scene’s boundaries aids in planning eye movements and other actions within the scene (Hubbard, 1996; Intraub, 2002). More recent theories have posited that boundary extension helps build an immersive egocentric spatial framework that integrates visual input from fixation to fixation (Intraub, 2012). These ideas, while attractive, are not congruent with boundary contraction. Other theories posit that those scene boundary transformations result from systematic memory biases toward the statistics of one’s visual experience (Bainbridge & Baker, 2020a; Bartlett, 1932; Hafri et al., 2022; Lin et al., 2022; Park et al., 2021). In other words, atypically, narrow-field views will extend in memory while larger-than-normal views will contract. Although there is some disagreement about whether these viewpoint statistics are category-specific (Bainbridge & Baker, 2020a; Lin et al., 2022; Park et al., 2021) or generalize to scenes as a whole (Hafri et al., 2022), this theoretical account is consistent with both boundary extension and contraction. It is worth noting that this view does not necessarily conflict with our information theoretic account. Photographs are acts of visual communication (Sontag, 2001). As such, the known viewpoint and compositional biases in photographs (Parkhurst & Niebur, 2003; Tatler et al., 2005) that lead to some viewpoints being more typical may reflect the desire of the photographer to optimize the amount of information that a viewer can apprehend at a glance.

We observed that memory for semantically sparse scenes was expanded while memory for semantically complex scenes contracted. This implies a cognitive process that normalizes the amount of semantic information in memory. Memories for scenes that exceed a semantic information capacity limit may be “zoomed in”, focusing primarily on central content as most of the informative content of a photograph is located there (Tatler, 2007). By contrast, semantically sparse scenes may be inflated via automatic inferences based on the scene gist (Friedman, 1979; Greene, 2016), or other prominent objects (Draschkow & Võ, 2017). These inferential processes may serve to guide subsequent saccades or other behavior (Intraub & Richardson, 1989), or may reflect mechanisms to improve the accuracy of noisy representations by leveraging prior knowledge (Hemmer & Steyvers, 2009; Kersten et al., 2004). This may be similar to other inferential processes that have been noted in boundary transformations. For example, scene boundary memories are biased toward the most canonical viewpoints of a given location (Lin et al., 2022).

Our results suggest that visual memory for scene boundaries is normalized towards a fixed capacity for semantic information. This is congruent with information-based capacity limitations for other forms of memory (Brady et al., 2009; Miller, 1956). For example, observers’ working memory capacities vary depending on the complexity of the objects held in memory (Alvarez & Cavanagh, 2004), suggesting that visual working memory has a fixed information capacity rather than a fixed capacity for objects or features. There is also evidence that visual experience can create compressed representations to increase working memory capacity. This has been shown through both training studies (Brady et al., 2009) and is reflected in the fact that working memory for real-world objects is higher than for abstract laboratory stimuli (Brady et al., 2016; Curby et al., 2009). This can be understood in a framework of semantic information. Familiar objects have consistent category labels (Murphy, 2004). This is a form of cognitive compression, allowing interlocutors to reason about a wide range of features that are associated with the category label. As there is a bidirectional interplay between categories and the features that give rise to them (Schyns, 1998), it is plausible that rare content leads observers to “zoom in” to gain the same amount of information that could have been offloaded to a category label, if one exists.

While accounts of capacity limitations speak to what can be lost in a memory representation, they cannot speak to what can be gained. Our memories are augmented through prior knowledge in the form of schemas (Bartlett, 1932). The question of what specific schematic knowledge may be filled in is open, but previous literature suggests several possibilities. First, we know that memory representations for specific scene details decay more quickly than memory for the gist (Zeng et al., 2021). Activating scene gist activates a set of probable objects (Friedman, 1979) shared across viewers (Greene, 2016). Further, we know that observers will often falsely remember seeing objects that are consistent with a particular scene gist (Brewer & Treyans, 1981; Castelhano & Henderson, 2008). Therefore, it may be the case that specific objects are expanded in cases with low semantic information. Another possibility is that the perception of some objects activates memory representations for other co-occurring objects (Koehler & Eckstein, 2017) and that these relations are expanded in memory. It seems likely that large anchor objects may drive the expansion of smaller, content objects (Draschkow & Võ, 2017). We believe that these possibilities are not mutually exclusive. Bayesian models of memory reconstruction have shown that prior knowledge in multiple hierarchical forms interacts (Hemmer & Steyvers, 2009). Thus, scene memory representations likely involve prior knowledge about objects, object relationships, viewpoints, and events.

Another open question is why the mind would be biased towards a fixed amount of semantic information rather than biased towards compressing memory whenever possible. The functional role of boundary extension has not yet been established (Bainbridge & Baker, 2020a, 2020b; Intraub, 2020). The classic view is that boundary extension enables the observer to make predictions about what is beyond the immediate view and to facilitate subsequent saccades (Intraub & Richardson, 1989). The idea that boundary transformations may serve predictive cognition is not incongruent with our view. Upon viewing a scene, the gist is activated (Oliva, 2005). This gist itself then activates the set of probable objects that could be found in the scene (Friedman, 1979; Greene, 2016). In this way, the gist increases the amount of semantic information, regardless of whether the predicted objects are fixated, or even if they are present (Brewer & Treyans, 1981).

The semantic information capacity limitation may also be related to previously described capacity limitations. Boundaries of images with highly emotional content (which are likely also images with a large amount of semantic information) have been shown to contract in memory (Takarangi et al., 2016) (although see Beighley et al., 2019). These types of images can also lead to an “attentional rubbernecking” effect that mimics the attentional blink (Most et al., 2005). It may be the case that images with highly emotional content have higher levels of semantic information as we define it. Let us consider two hypothetical images: a man holding a cell phone and a man holding a gun. We posit that the descriptions of the latter picture may center around the gun, the man’s motivations for holding it, and so on. By contrast, the act of holding a cell phone is so common as not even to warrant a mention. Therefore, the first picture would generate descriptions with fewer words, and thus contain less semantic information than the second. Finally, it may also be the case that images with a large amount of semantic information also impair early visual perception: images that depicted low-probability real-world events led to lower detection and categorization performance compared to perceptually-matched images of more probable content (Greene et al., 2015). In this study, observers generated descriptions of probable and improbable images that were presented for varying durations ranging from 24 ms to unlimited. It is worth noting that the descriptions of improbable images in the unlimited viewing duration were longer than those of the matched probable images and thus would register as higher semantic information using the metrics in the present study.

We did not necessarily expect that memory for scene boundaries did not show a fixed information limit for visual information. Previous work has shown that images with high levels of visual information (indexed by similar features to the ones used here) led to less efficient visual search and lower performance on target detection in low contrast (Rosenholtz et al., 2007). It may be the case that while ongoing visual processes such as visual search are limited by visual information, scene memory representations are more conceptual and thus limited by semantic information. It could also be the case that we saw no effect of visual information because we observed a quadratic relationship between our visual metrics and scene depth. Scenes with a mid-level depth had the most visual information, while very shallow and deep scenes had lower visual information. This is congruent with previous results that show systematic differences in specific image statistics, such as the slope of the amplitude spectrum, with scene depth (Torralba & Oliva, 2003). Our metrics of visual information are also related to these lower-level image statistics. Thus, visual information may play a nonlinear role in the memory for scene boundaries. This possibility is congruent with the findings of (Sun & Firestone, 2022) who found that visual stimuli of medium visual complexity led to longer verbal descriptions than images with lower or higher complexity levels. A final possibility is that the three metrics of visual information that we used, although motivated by previous literature, may not fully tap into biologically and psychologically relevant features. Future work may expand the number of visual features to include other metrics that have been shown to influence behavioral and neural responses, such as Weibull statistics of oriented filters (Groen et al., 2012).

While our results demonstrate that memory errors for scene boundaries can be predicted by each image’s semantic information, much more predictive power is provided by scene depth. However, combining both depth and semantic information allows us to explain about 90% of explainable variability in scene boundary transformation scores. Thus, semantic information provides a critical element in the memory representation of scenes.

## ACKNOWLEDGMENTS

We would like to thank Alon Hafri and one anonymous reviewer for helpful feedback. Thanks to Wilma Bainbridge and Lindsay Houck for useful discussions of this work.

## AUTHOR CONTRIBUTIONS

Michelle R. Greene: Conceptualization, Methodology, Project Administration, Supervision, Visualization, Writing. Devanshi Trivedi: Formal Analysis, Methodology, Software, Visualization, Writing.

## FUNDING INFORMATION

This project was supported by the Bates College Program in Neuroscience.

## DATA AVAILABILITY STATEMENT

Data and stimuli are available at https://osf.io/wn7v2/.
